# Identification and differential expression analysis of anthocyanin biosynthetic genes in leaf color variants of ornamental kale

**DOI:** 10.1186/s12864-019-5910-z

**Published:** 2019-07-08

**Authors:** Ning Guo, Shuo Han, Mei Zong, Guixiang Wang, Shuning Zheng, Fan Liu

**Affiliations:** 1 Beijing Vegetable Research Center, Beijing Academy of Agriculture and Forestry Sciences, National Engineering Research Center for Vegetables, Key Laboratory of Biology and Genetic Improvement of Horticultural Crops (North China), Ministry of Agriculture, P. R. China, Beijing, 100097 China; 2 Institute of Vegetables and Flowers, Chinese Academy of Agricultural Sciences, Beijing, 100081 China

**Keywords:** Anthocyanin accumulation, Anthocyanin biosynthetic genes, *Brassica oleracea*, Color variants, Differentially expressed genes, Ornamental kale, Regulatory network

## Abstract

**Background:**

Anthocyanins perform diverse biological functions in plants and are beneficial to human health. Leaf color is the most important trait of ornamental kale and the characteristics of changes in leaf color make it an ideal material to elucidate genetic mechanisms of anthocyanins accumulation in *Brassica oleracea*. To elucidate the anthocyanin distribution, metabolic profiles and differentially expressed anthocyanin biosynthetic genes between different colored accessions can pave the way for understanding the genetic regulatory mechanisms of anthocyanin biosynthesis and accumulation in ornamental kale.

**Results:**

In this study, anthocyanin distributions in red- and white-leaved ornamental kale accessions were determined. Thirty-four anthocyanins were detected in the red-leaved accession. The complete set of anthocyanin biosynthetic genes in the *B. oleracea* reference genome was identified and differential expression analysis based on RNA-seq was conducted. Eighty-one anthocyanin biosynthetic genes were identified in the *B. oleracea* reference genome. The expression patterns and differential expressions of these genes in different leaf types indicated that late biosynthetic genes (*BoDFR1*, *BoANS1* and *2*, and *BoUGT79B1.1*), positive regulatory genes (*BoTTG1*, *BoTT8*, and *Bol012528*), a negative regulatory gene (*BoMYBL2.1*), and transport genes (*BoTT19.1* and *BoTT19.2*) may play roles in anthocyanin accumulation in ornamental kale. A genetic regulatory network of anthocyanin accumulation in ornamental kale was constructed.

**Conclusions:**

The distribution of pigments and anthocyanin profiles explained the leaf color phenotypes of ornamental kales. The identification of key genes and construction of genetic regulatory network in anthocyanin accumulation in ornamental kale elucidated the genetic basis of leaf color variants. These findings enhance the understanding of the genetic mechanisms and regulatory network of anthocyanin accumulation in *B. oleracea*, and provide a theoretical basis for breeding new cultivars of *Brassica* vegetables with enhanced ornamental and nutritional value.

**Electronic supplementary material:**

The online version of this article (10.1186/s12864-019-5910-z) contains supplementary material, which is available to authorized users.

## Background

The colors of leaves, flowers and fruits of agricultural products are highly important for their commercial value. Anthocyanins, which are water-soluble pigments synthesized in many plants, are an important type of flavonoid compound that contribute to the majority of the orange, red, blue, and purple colors. Anthocyanins perform extremely diverse functions in plants. They serve as attractants of pollinators and seed dispersers, and play important roles in responses to abiotic and biotic stresses [33]. Anthocyanins also have beneficial roles in human health. As potent antioxidants, they are potentially protective against cardiovascular disease, certain cancers, and some other chronic diseases [4, 13, 21]. Therefore, a comprehensive understanding of anthocyanin biosynthesis is important for development of foods that are rich in anthocyanins to meet the increasing demand for health-promoting components in our daily diet.

The biosynthetic pathways of anthocyanins have been well characterized [12] and the corresponding genes have been isolated from various plant species. In the model plant *Arabidopsis thaliana*, the biosynthesis, regulation, and transport of anthocyanins, specifically the majority of the structural and regulatory genes involved in anthocyanin synthesis, have been identified and functionally characterized in the last two decades [3, 16, 38]. These studies have made important contributions to the comprehensive understanding of anthocyanin biosynthesis and have revealed the accumulation and metabolic profiles of anthocyanins in *Arabidopsis*.

Anthocyanins are derived from branches of the flavonoid pathway, which starts with phenylalanine via the general phenylpropanoid pathway. The phenylpropanoid pathway contains three major genes: *PAL* (phenylalanine ammonia-lyase), *C4H* (cinnamate-4-hydroxylase) and *4CL* (4-coumarate: CoA ligase). Two types of correlated structural genes can be distinguished in the flavonoid biosynthetic pathway: early biosynthetic genes (EBGs) and late biosynthetic genes (LBGs) [26]. The EBGs, which include *CHS* (chalcone synthase), *CHI* (chalcone isomerase), *F3H* (flavanone 3-hydroxylase), *F3′H* (flavonoid 3′-hydroxylase), and *FLS* (flavonol synthase), lead to the production of flavonols and other flavonoid compounds, whereas the LBGs, which include *DFR* (dihydroflavonol-4-reductase), *ANS* (anthocyanidin synthase), and *UFGT* (UDP-glucose: flavonoid 3-*O*-glucosyltransferase), lead to the production of anthocyanins [16].

At the transcription level anthocyanin biosynthesis is mainly regulated by a series of transcription factors, especially members of the R2R3-MYB gene family. While EBGs are activated by co-activator-independent and functionally redundant R2R3-MYB genes (e.g., *MYB11*, *MYB12*, and *MYB111* in *Arabidopsis*), LBGs are activated by a highly conserved MYB–bHLH–WD40 (MBW) transcriptional activation complex ([11, 26, 41]; Xu et al., 2015 [40]). In *Arabidopsis*, the R2R3-MYB genes *PAP1*, *PAP2*, *MYB113*, and *MYB114* [2, 8], the bHLH gene family members *TT8*, *GL3*, and *EGL3* [23, 25, 42], and the WD40 family gene *TTG1* [37] are recognized as the key genes encoding respective components of the MBW complex. In addition, two single-repeat R3-MYB transcription factors, *MYBL2* ([6]a; [22]) and *CPC* (CAPRICE) [43], and three members of the LATERAL ORGAN BOUNDARY DOMAIN (LBD) gene family, *LBD37*, *LBD38*, and *LBD39* [29], are negative regulators of anthocyanin biosynthesis in *Arabidopsis*.

Anthocyanins are synthesized on the cytosolic surface of the endoplasmic reticulum (ER) but predominantly accumulate in the vacuole. Transparent Testa 19 (TT19), a glutathione S-transferase (GST), which functions as a carrier to transport anthocyanins to the tonoplast, plays a key role in anthocyanins transportation and accumulation. TT19 binds to the anthocyanins synthesized on the cytosolic surface of the ER. The pigment–TT19 binary complex in the cytosol is recruited to the tonoplast. The pigments may be acylated, prior to release from TT19, and subsequently sequestered into vacuoles by transporters [32].

Numerous vegetable crops derived from *Brassica oleracea* are important worldwide, including cauliflower, broccoli, cabbage, Brussels sprouts, kohlrabi, and kale. Almost all cultivar groups include red- or purple-pigmented types, in which anthocyanins are deposited in various organs, especially the leaves, stems, siliques, and curds [27]. Ornamental kale (*B. oleracea* var. *acephala*), which is an excellent ornamental foliage plant with a range of leaf colors and shapes, is widely cultivated as a landscape plant, potted plant, and cut flower. It is sufficiently tolerant of frost and chilling that it can grow vigorously in regions that experience low temperature [17]. The variety of leaf colors is the most typical characteristic of ornamental kale. The outer, mature leaves are green, whereas the central (newly developed) leaves may be white, pink, red, or purple. The color of the new leaves is determined by the content of anthocyanins. Leaf color is the most important trait of kale for its ornamental value and nutritional quality, as well as cold resistance.

Some leaf color-related candidate genes have been mapped in ornamental kale. For example, the red leaf color trait is reportedly controlled by the single dominant gene *Re* in ornamental kale, which was mapped to chromosome C09 [28]. Zhu et al. determined the pink leaf color trait in ornamental kale to be controlled by a single semi-dominant gene mapped to chromosome C03 [44]. *BoPr*, which was identified as a gene encoding a dihydroflavonol reductase (DFR), was mapped on chromosome C09 and controls the purple leaf trait of ornamental kale [20]. The change in color of the inner leaves (from green to red or white) of ornamental kales is induced by low temperature, and thus may be a type of low temperature adaptation. The mechanism underlying such color formation remains poorly understood. A complete understanding of the structural and regulatory genes involved as well as analysis of the differential expression patterns in young and mature leaves and among cultivars is important for elucidation of the mechanism of anthocyanin biosynthesis and leaf pigmentation in ornamental kale.

In a previous study, the anthocyanin biosynthetic genes in *B. rapa* (BrABGs) were identified and analyzed by comparative genomic analysis [9]. In the present study, the anthocyanin profile was determined in red ornamental kale using liquid chromatography–mass spectrometry (LC–MS), the complete set of anthocyanin biosynthetic genes in *B. oleracea* was identified by comparative genomic analysis, and RNA sequencing (RNA-seq) of central (new) and outer (mature) leaves from red- and white-leaved ornamental kales was conducted to analyze the differential expression of anthocyanin biosynthetic genes. The results of our studies will advance the understanding of the mechanism of anthocyanin biosynthesis at the gene expression level, and provide a foundation for further cultivar improvement and the breeding of novel cultivars of enhanced ornamental and nutritional value.

## Results

### Anthocyanin distribution in red- and white-leaved ornamental kales

**Fig. 1:**
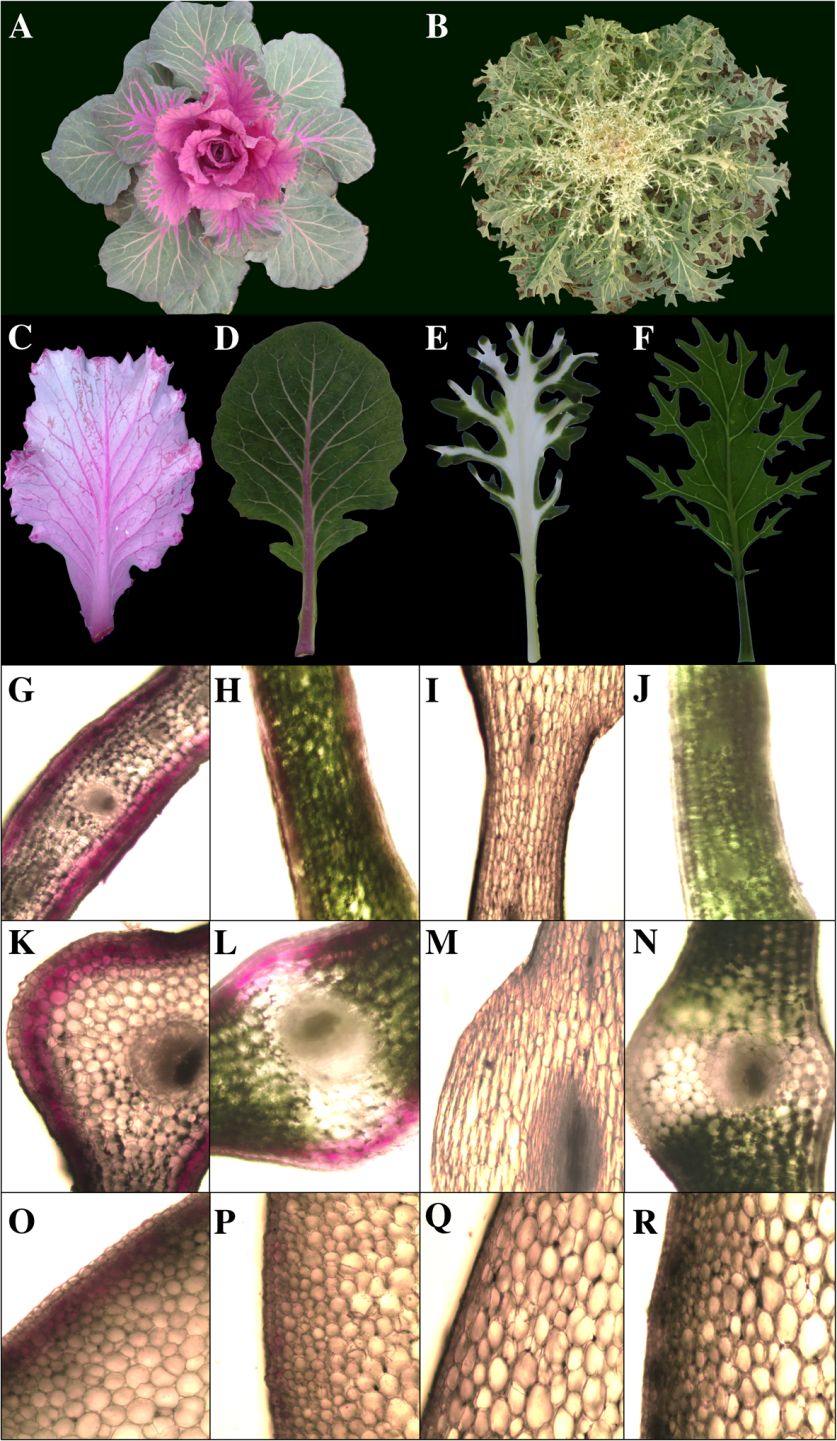
Individual plants, new and mature leaves, and anatomical distribution of leaf pigments in ornamental kale DH lines ‘05-DH-65’ and ‘06-DH-71’. (**a**) ‘05-DH-65’ plant; (**b**) ‘06-DH-71’ plant; (**c**) ‘05-DH-65’ new (central) leaf; (**d**) ‘05-DH-65’ mature (outer) leaf; (**e**) ‘06-DH-71’ new (central) leaf; (**f**) ‘06-DH-71’ mature (outer) leaf; (**g**) ‘05-DH-65’ new leaf lamina, transverse section; (**h**) ‘05-DH-65’ mature leaf lamina, transverse section; (**i**) ‘06-DH-71’ new leaf lamina, transverse section; (**j**) ‘06-DH-71’ mature leaf lamina, transverse section; (**k**) ‘05-DH-65’ new leaf vein, transverse section; (**l**) ‘05-DH-65’ mature leaf vein, transverse section; (**m**) ‘06-DH-71’ new leaf vein, transverse section; (**n**) ‘06-DH-71’ mature leaf vein, transverse section; (**o**) ‘05-DH-65’ new leaf petiole, longitudinal section; (**p**) ‘05-DH-65’ mature leaf petiole, longitudinal section; (**q**) ‘06-DH-71’ new leaf petiole, longitudinal section; (**r**) ‘06-DH-71’ mature leaf petiole, longitudinal section

We observed that the ornamental kales began to show color development, i.e. the newly developing leaves became red or white, when the minimum temperature decreased below 10 °C. For the red-leaved DH line ‘05-DH-65’, the central new leaves are purple-red (Fig. 1c), the petiole and veins of the outer, mature leaves are red, whereas the remainder of the leaf lamina is green (Fig. 1d). For the white-leaved DH line ‘06-DH-71’, the central new leaves are almost white (Fig. 1e) and the outer mature leaves are green (Fig. 1f). Leaf anatomical observations revealed that in the lamina of new leaves of ‘05-DH-65’, red-purple pigmentation was present in the adaxial and abaxial epidermal cells as well as one to several adjacent cell layers of the palisade or spongy mesophyll, whereas internal mesophyll cells were transparent and contained almost no pigments, including chlorophylls (Fig. 1g). The transverse and longitudinal sections of the veins and petiole of new leaves of ‘05-DH-65’ revealed an identical distribution of pigments to that of the lamina. With regard to mature leaves of ‘05-DH-65’, the distribution of red-purple pigments was identical to that of new leaves; however, red-purple pigments were less abundant in mature leaves and the internal leaf mesophyll cells, except in the veins and petioles, contained chlorophylls and appeared green, while the veins and petiole were red-purple (Fig. 1d, h, l, p). Sections of new leaves of ‘06-DH-71’ revealed that almost no pigments were present in all cells (Fig. 1I, m, q). Mature leaves of ‘06-DH-71’ contained chlorophylls but no red pigments (Fig. 1j, n, r).

### Anthocyanins profile and content analysis

**Fig. 2:**
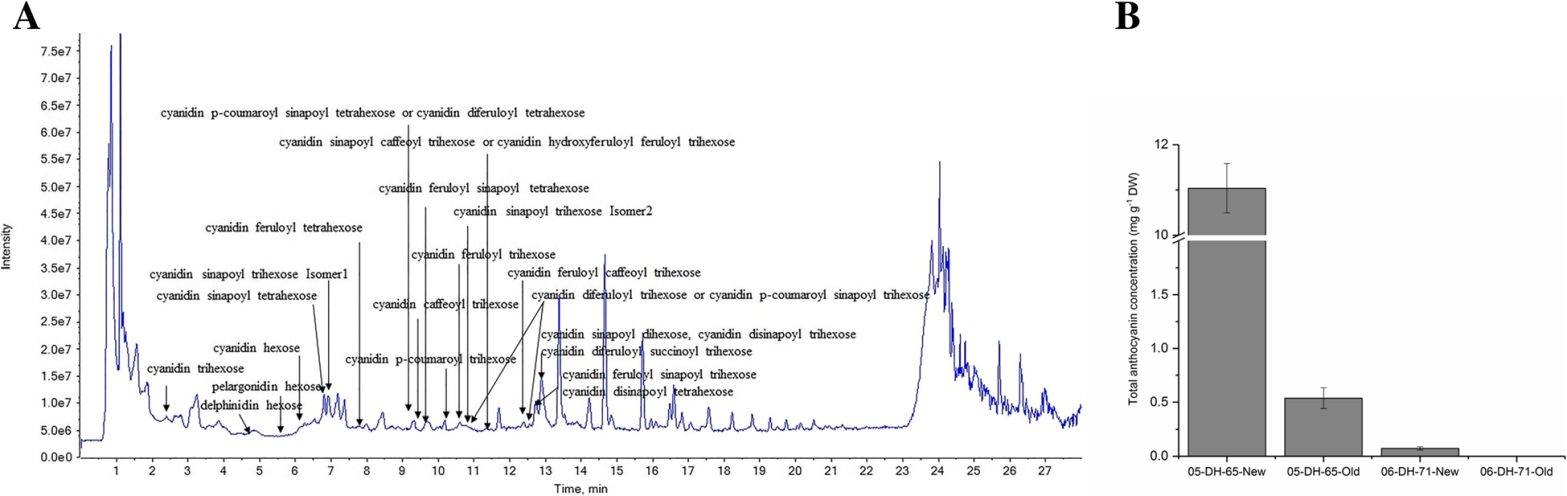
**a** Liquid chromatography chromatograms (520 nm) of anthocyanin extracts from new leaves of the ornamental kale DH line ‘05-DH-65’. *Horizontal axis* shows retention time (min); *vertical axis* shows the strength of the chromatographic peak response intensity. The compound name is provided for each peak. **b** Total anthocyanins content in new and mature leaves of the DH lines ‘05-DH-65’ and ‘06-DH-71’

The red-purple pigments in ornamental kale are due to anthocyanins. To characterize the anthocyanin profile, we performed UHPLC-TOF analysis of new leaves of the red-leaved DH line ‘05-DH-65’. In total, 34 anthocyanin compounds were detected (Fig. 2a; Table 1). Identification of anthocyanins in extracts was based on a method combining chromatographic behavior, accurate molecular mass spectra obtained by Q-TOF mass spectrometry, characteristic MS/MS fragmentation product ions obtained by Q-Trip mass spectrometry, UV spectra, and comparison with previous publications [10, 18, 39]. The glycosyl groups were hexoses, which were mainly glucosides, and ranged in the number from one to four. The glycosyl groups were acylated by sinapoyl, *p*-coumaroyl, caffeoyl, feruloyl, and hydroxyferuloyl. Several isomers with an identical formula were detected, thus the glycosyl groups may be located in different positions. A comprehensive metabolic profile of anthocyanins in ‘05-DH-65’ was generated (Table 1). The TAC in new and mature leaves of ‘05-DH-65’ and ‘06-DH-71’ were determined by pH differential spectrophotometry. The TACs in new and mature leaves of ‘05-DH-65’ were 11.04 and 0.54 mg/g DW, respectively, whereas the TACs in new and mature leaves of ‘06-DH-71’ were 0.073 and 0 mg/g DW, respectively (Fig. 2b).

**Table 1:** UHPLC-PDA-Q-TOF data and putative identification of anthocyanins from new leaves of the ornamental kale DH line ‘05-DH-65’

Anthocyanins	Formula	*t*_R_ (min)^a^	Mass (Da)	Major and important productions (m/z)
Cyanidin hexose Isomer1	C21H21O11	6.16	449.11	287.06
Cyanidin hexose Isomer2	C21H21O11	7.37	449.11	287.06
Cyanidin hexose Isomer3	C21H21O11	12.37	449.11	287.05
Cyanidin hexose Isomer4	C21H21O11	12.57	449.11	287.06
Cyanidin dihexose Isomer1	C27H31O16	6.12	611.16	449.10, 287.05
Cyanidin dihexose Isomer2	C27H31O16	6.35	611.16	287.05
Cyanidin trihexose Isomer1	C33H41O21	2.46	773.21	611.16, 449.11, 287.06
Cyanidin trihexose Isomer2	C33H41O21	6.11	773.21	611.16, 449.11, 287.05
Cyanidin sinapoyl dihexose	C38H41O20	12.90	817.22	655.16, 499.11, 287.06
Cyanidin p-coumaroyl trihexose	C42H47O23	10.24	919.25	757.19, 49.11, 287.05
Cyanidin caffeoyl trihexose	C42H47O24	9.17	935.25	773.20, 449.11, 287.05
Cyanidin tetrahexose Isomer1	C39H51O26	6.35	935.27	773.21, 611.16, 449.11, 287.05
Cyanidin tetrahexose Isomer2	C39H51O26	7.47	935.27	773.22, 611.16, 449.11, 287.06
Cyanidin feruloyl trihexose	C43H49O24	10.65	949.26	787.21, 449.11, 287.06
Cyanidin hydroxyferuloyl trihexose	C43H49O25	6.47	965.26	803.21, 449.11, 287.05
Cyanidin sinapoyl trihexose Isomer1	C44H51O25	6.92	979.27	817.22, 449.11, 287.06
Cyanidin sinapoyl trihexose Isomer2	C44H51O25	10.81	979.27	817.22, 449.11, 287.06
Cyanidin feruloyl tetrahexose	C49H59O29	7.75	1111.31	949.26, 449.11, 287.05
Cyanidin feruloyl caffeoyl trihexose	C52H55O27	12.42	1111.29	949.24, 449.11, 287.05
Cyanidin diferuloyl trihexose or Cyanidin p-coumaroyl sinapoyl trihexose Isomer1	C53H57O27	10.73	1125.31	963.24, 449.11, 287.05
Cyanidin diferuloyl trihexose or Cyanidin p-coumaroyl sinapoyl trihexose Isomer2	C53H57O27	12.39	1125.31	963.26, 449.11, 287.06
Cyanidin sinapoyl tetrahexose	C50H61O30	6.91	1141.32	979.27, 817.21, 449.11, 287.05
Cyanidin sinapoyl caffeoyl trihexose or Cyanidin hydroxyferuloyl feruloyl trihexose	C53H57O28	11.29	1141.30	979.25, 449.11, 287.06
Cyanidin feruloyl sinapoyl trihexose	C54H59O28	12.73	1155.32	993.27, 449.11, 287.06
Cyanidin disinapoyl trihexose	C55H61O29	12.90	1185.33	1023.28, 449.1077;287.0573
Cyanidin diferuloyl succinoyl trihexose	C57H61O30	12.93	1225.32	1045.27, 759.22, 287.06
Cyanidin p-coumaroyl sinapoyl tetrahexose or Cyanidin diferuloyl tetrahexose	C59H67O32	9.44	1287.36	1225.31, 449.11, 287.05
Cyanidin feruloyl sinapoyl tetrahexose	C60H69O33	9.75	1317.37	1155.32, 449.11, 287.06
Cyanidin disinapoyl tetrahexose	C61H71O34	12.88	1347.38	1185.33, 449.09, 287.06
Pelargonidin hexose	C21H21O10	5.68	433.11	271.06
Delphinidin hexose Isomer1	C21H21O12	4.82	465.10	303.05
Delphinidin hexose Isomer2	C21H21O12	6.81	465.10	303.05
Delphinidin hexose Isomer3	C21H21O12	10.44	465.10	303.05
Delphinidin hexose Isomer4	C21H21O12	11.84	465.10	303.05

^a^ *t*_R_: retention time

### Identification, chromosomal localization and subgenomic distribution of anthocyanin biosynthetic genes

Forty-one anthocyanin biosynthetic genes (ABGs) have been identified in the *Arabidopsis* genome, consisting of 24 structural genes encoding anthocyanin biosynthetic enzymes, 16 regulatory genes encoding transcriptional factors, and one transport gene that is required for anthocyanin transportation (Table 2). Based on a combination of syntenic and non-syntenic homology analysis, 81 *B. oleracea* anthocyanin biosynthetic genes (BoABGs) were identified, representing homologs for 39 of the 41 AtABGs; homologs of two AtABGs (*AtFLS6* and *AtMYB11*) were not detected in *B. oleracea*. Among the 81 BoABGs, 57 were syntenic orthologs and 24 were non-syntenic homologs. The major events responsible for BoABGs copy number expansion were whole genome duplication (WGD) and tandem duplication (TD). Nine AtABGs had two syntenic orthologs and an additional nine AtABGs had three syntenic orthlogs. The BoABGs had less than three syntenic orthologs as a result of gene fractionation that occurred following the WGT. Some AtABGs were TD genes that formed a gene cluster, such as *AtFSL2–5*, four FSL-encoding genes, and three *R2R3-MYB* genes (*AtPAP1*, *AtMYB113*, and *AtMYB114*) that form the MBW complex to regulate LBGs; their syntenic *B. oleracea* orthologs had both lost the TD paralogs. However, some BoABGs, especially structural genes, had undergone TD to form gene clusters, such as *Bo4CL5.1* to *5.5*, *BoF3H4* and *BoF3H5*, *BoFLS1.2* and *BoFLS1.3*, and *BoUGT79B1.2* to *1.5*. These TDs were all novel in *B. oleracea*, which represented an important means to increase gene number. For example, syntenic orthologs of *At4CL5* were lacking in *B. oleracea*, whereas non-syntenic orthologs formed a tandem cluster of five genes. The same pattern was observed for *AtUGT79B1*, which did not experience WGD but its non-syntenic orthologs were duplicated by TD.

**Table 2:** Anthocyanin biosynthetic genes identified in *B. oleracea* by comparative genomic analysis with the *Arabidopsis thaliana* genome

	*B. oleracea*			
*A. thaliana*	Synteny orthologs			Non-synteny orthologs
	LF	MF1	MF2	
Structural genes				
*Biosynthetic genes in phenylpropanoid pathway*				
*AtPAL1* (AT2G37040)	***BoPAL1.1*** (Bol025522)	***BoPAL1.2*** (Bol037689)		
*AtPAL2* (AT3G53260)	***BoPAL2.1*** (Bol025102)	***BoPAL2.2*** (Bol041738)	***BoPAL2.3*** (Bol005411)	***BoPAL2.4*** (Bol005084)
*AtPAL3* (AT5G04230)	–	–	***BoPAL3.1*** (Bol005493)	***BoPAL3.2*** (Bol006745)
*AtPAL4* (AT3G10340)	***BoPAL4*** (Bol011375)	–	–	–
*AtC4H* (AT2G30490)	–	***BoC4H1*** (Bol033347)	***BoC4H3*** (Bol004608)	***BoC4H5*** (Bol006704)
		***BoC4H2*** (Bol033349)	***BoC4H4*** (Bol004610)	
*At4CL1* (AT1G51680)	–	–	***Bo4CL1*** (Bol031583)	–
*At4CL2* (AT3G21240)	–	–	–	***Bo4CL2.1*** (Bol038385)
				***Bo4CL2.2*** (Bol038386)
*At4CL3* (AT1G65060)	***Bo4CL3*** (Bol012584)	–	–	–
*At4CL5* (AT3G21230)	–	–	–	***Bo4CL5.1*** (Bol000926)
				***Bo4CL5.2*** (Bol038387)
				***Bo4CL5.3*** (Bol038389)
				***Bo4CL5.4*** (Bol026622)
				***Bo4CL5.5*** (Bol026623)
*Early biosynthetic genes*				
*AtCHS* (AT5G13930)	***BoCHS1*** (Bol043396)	***BoCHS2*** (Bol034259)	***BoCHS3*** (Bol004244)	
*AtCHI* (AT3G55120)	***BoCHI1*** (Bol044343)	***BoCHI2*** (Bol044344)	***BoCHI3*** (Bol008652)	***BoCHI4*** (Bol018696)
*AtF3H* (AT3G51240)	***BoF3H1*** (Bol010585)	***BoF3H2*** (Bol030864)	***BoF3H3*** (Bol002277)	***BoF3H4*** (Bol044664)
				***BoF3H5*** (Bol041656)
*AtF3’H* (AT5G07990)	***BoF3’H*** (Bol043829)			
*AtFLS1* (AT5G08640)	***BoFLS1.1*** (Bol043773)			***BoFLS1.2*** (Bol004505)
				***BoFLS1.3*** (Bol019362)
*AtFSL2* (AT5G63580) *AtFLS3* (AT5G63590)			***BoFLS2*** (Bol019125)	
*AtFLS4* (AT5G63595)				
*AtFLS5* (AT5G63600)				
*AtFLS6* (AT5G43935)				
*Late biosynthetic genes*				
*AtDFR* (AT5G42800)			***BoDFR1*** (Bol035269)	***BoDFR2*** (Bol006005)
*AtANS* (AT4G22880)	***BoANS1*** (Bol014986)	***BoANS2*** (Bol042059)		
*AtUGT79B1* (AT5G54060)	***BoUGT79B1.1*** (Bol038805)			***BoUGT79B1.2*** (Bol014515)
				***BoUGT79B1.3*** (Bol014517)
				***BoUGT79B1.4*** (Bol014519)
				***BoUGT79B1.5*** (Bol018228)
*AtUGT75C1* (AT4G14090)			***BoUGT75C1*** (Bol027055)	
*AtUGT78D2* (AT5G17050)			***BoUGT78D2*** (Bol021317)	
Regulatory genes (Transcription factor)				
Positive regulators				
*R2R3-MYB*				
*Independent regulatory genes*				
*AtMYB11* (AT3G62610)				
*AtMYB12* (AT2G47460)	***BoMYB12.1*** (Bol001533)	***BoMYB12.2*** (Bol002581)	***BoMYB12.3*** (Bol029626)	
*AtMYB111* (AT5G49330)	***BoMYB111.1*** (Bol016599)	***BoMYB111.2*** (Bol033054)	***BoMYB111.3*** (Bol032351)	
*Regulation by forming MBW complex*				
*AtPAP1* (AT1G56650)				
*AtPAP2* (AT1G66390)				Bol042409 Bol012531
*AtMYB113* (AT1G66370)	Bol012528	Bol045347		
*AtMYB114* (AT1G66380)				
*bHLH*				
*AtTT8* (AT4G09820)	***BoTT8*** (Bol004077)			
*AtGL3* (AT5G41315)	***BoGL3*** (Bol014556)			
*AtEGL3* (AT1G63650)		***BoEGL3.1*** (Bol022614)	***BoEGL3.2*** (Bol029662)	***BoEGL3.3*** (Bol004759)
*WD40*				
*AtTTG1* (AT5G24520)	***BoTTG1*** (Bol022420)			
Negative regulators				
*Single-Repeat R3 MYB*				
*AtMYBL2* (AT1G71030)	***BoMYBL2.1*** (Bol016164)	***BoMYBL2.2*** (Bol034966)		
*AtCPC* (AT2G46410)	***BoCPC1*** (Bol000928)	***BoCPC2*** (Bol021780)	***BoCPC3*** (Bol029590)	
*LATERAL ORGAN BOUNDARY DOMAIN (LBD)*				
*AtLBD37* (AT5G67420)	***BoLBD37.1*** (Bol014304)	***BoLBD37.2*** (Bol008082)	***BoLBD37.3*** (Bol005707)	
*AtLBD38* (AT3G49940)	***BoLBD38.1*** (Bol007980)	***BoLBD38.2*** (Bol021982)	***BoLBD38.3*** (Bol016975)	
*AtLBD39* (AT4G37540)				***BoLBD39*** (Bol019060)
Transport genes				
*AtTT19* (AT5G17220)	***BoTT19.1*** (Bol019821)		***BoTT19.2*** (Bol021325)	

**Fig. 3:**
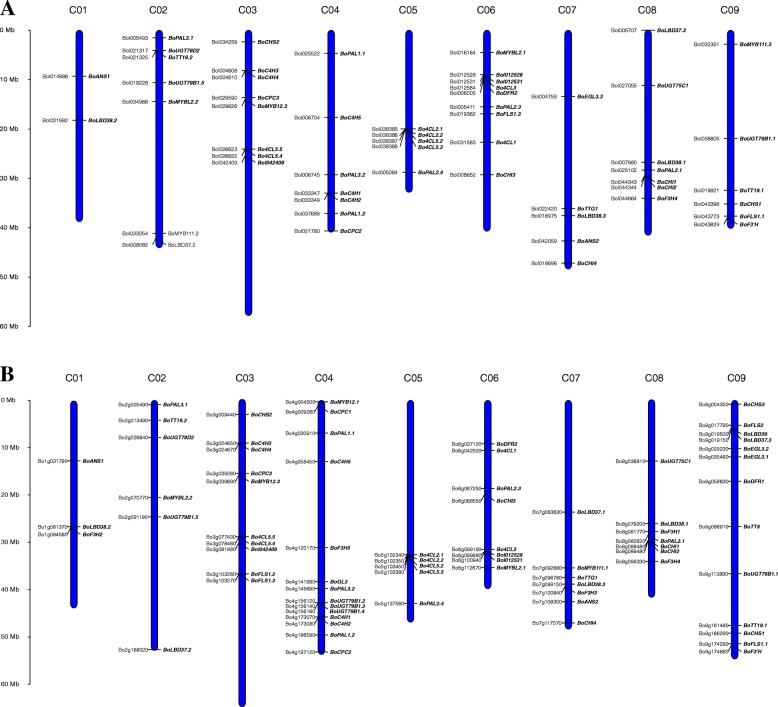
Distribution of anthocyanin biosynthetic genes (ABGs) on the pseudo-chromosomes of *B. oleracea* reference genome versions Cap02–12 (**a**) and TO1000 (**b**). The blue bars represent the nine chromosomes (C01 to C09) of *B. oleracea*. The relative positions of *BoABGs* are marked on the pseudo-chromosomes of genome versions Cap02–12 (**a**) and TO1000 (**b**). Gene annotations and names are provided on the left and right sides of the bars, respectively. The scale indicates the physical distance of the chromosomes

Of the 81 BoABGs, 56 were mapped to the nine chromosomes of *B. oleracea*. Thus, 2, 7, 8, 7, 5, 9, 5, 7, and 6 BoABGs were located on chromosomes C01 to C09, respectively, of *B. oleracea* genome V1.1 (Cap 02–12) (Fig. 3a). A higher number of BoABGs (73) were mapped on the genome V2.1 (TO1000) [24] (Fig. 3b), with 3, 6, 10, 14, 5, 8, 7, 7, and 13 located on chromosomes C01 to C09, respectively. However, the distributions of the genes differed between the two reference genome versions. The corresponding gene IDs of ABGs in the two genome versions are listed in Additional file 7: Table S1.

### Overall expression of genes in the anthocyanin biosynthesis pathway

From mRNA sequencing, raw data were obtained from three biological repeats for new and mature leaves of the red- and white-leaved DH lines ‘05-DH-65’ and ‘06-DH-71’. The summary statistics of the RNA sequencing was showed in Additional file 7: Table S2. Gene expression levels were calculated using the expected FPKM values. The expression values distribution of different samples were showed in Additional file 1: Figure S1A which demonstrated the overall expression levels among different samples. In order to validate these biological replicates sampled with good agreement replicates, these samples were clustered by gene expression values between each other (Additional file 1: Figure S1B). From these results we could clearly see that the three biological repeats were clustered together indicating good repeatability.

Of the 81 BoABGs, expression of 15 genes was not detected and six genes showed extremely low expression levels (FPKM value < 1) in all of the new and mature leaves of ‘05-DH-65’ and ‘06-DH-71’. These genes were identified as duplicated paralogs that arose by WGD or TD, and at least one of the paralogs was expressed. For example, of the five identified flavanone 3-hydroxylase (F3H) coding genes, *BoF3H1* to *5*, only *BoF3H1* was expressed in new and mature leaves of ‘05-DH-65’ and ‘06-DH-71’, whereas expression of the other four paralogs was not detected. *BoDFR2* was not expressed in each of the four leaf types, whereas its paralog *BoDFR1* was differentially expressed in new and mature leaves of the two accessions. Some genes were especially expressed in ‘05-DH-65’, such as *Bo4CL2.1* and *BoUGT79B1.1*, whereas the negative regulatory gene *BoMYBL2.1* was only expressed in ‘06-DH-71’. The expression FPKM values are listed in Additional file 7: Table S3.

To determine the expression patterns of BoABGs, a heatmap combined with a hierarchical clustering analysis was constructed based on gene expression (FPKM values were log2 transformed) levels in new and mature leaves of ‘05-DH-65’ and ‘06-DH-71’. The expression levels of BoABGs varied among the different leaves (Additional file 2: Figure S2). Anthocyanin biosynthetic genes from new and mature leaves of the same accession were clustered in the same group based on the BoABGs expression levels. Thus, BoABGs showed different expression patterns in the red- and white-leaved accessions. The 81 BoABGs were clustered into two main groups. The first group contained five genes (*BoCHS1*, *2*, *3*, *BoF3H1*, and *BoFLS1.1*) that were expressed at relatively higher levels in all of the four leaf types. These genes were all EBGs that lead to the production of flavonols and other flavonoid compounds. The second group comprised two subgroups. In the first subgroup were the genes expressed at low levels in almost all of the four leaf types. As already mentioned, these genes were mainly paralogs that were expanded by WGD or TD. The genes in the second subgroup showed differential expression in the four leaf types. *BoDFR1*, *BoANS1*, *BoANS2*, *BoUGT75C1*, and *BoUGT79B1*, which are all LBGs, were most highly expressed in new leaves of ‘05-DH-65’ and showed the lowest expression level in mature leaves of ‘06-DH-71’. The transport genes *BoTT19.1* and *BoTT19.2* showed an identical expression pattern.

### The expression analysis of genes in the anthocyanin biosynthetic pathway among different leaf types

**Fig. 4:**
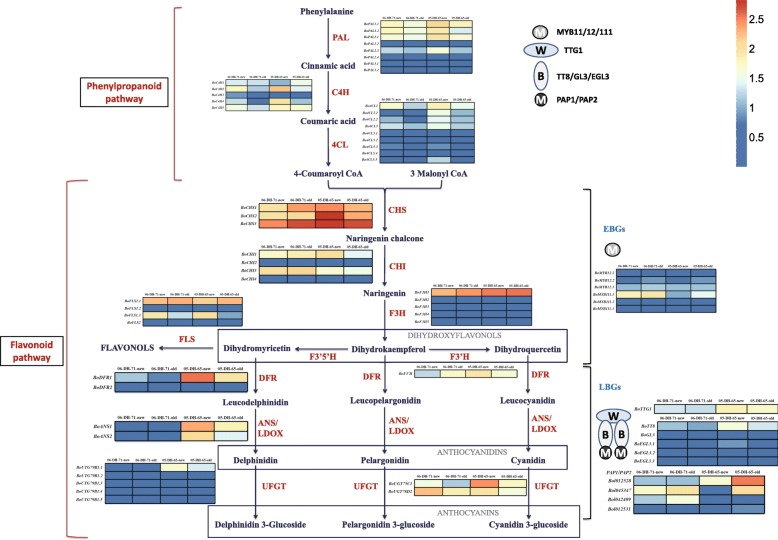
The anthocyanin biosynthetic pathway and expression levels of structural and positive regulatory genes in *B. oleracea*. The pathway can be divided into two sections: the phenylpropanoid and the flavonoid pathways. Two types of genes are involved in the flavonoid pathway: early biosynthetic genes (EBGS) and late biosynthetic genes (LBGs). Red type indicates biosynthetic enzymes. The ellipses “M”, “B”, and “W” represent the MYB, bHLH, and WD40 proteins involved in the positive regulation of anthocyanin synthesis. The expression levels of coding genes is indicated by blue and red shading, which represents low to high expression levels, respectively. The color scale corresponds with the mean-centered log2-transformed FPKM values, which are identical to those in Fig. 4

The variation in expression of structural and regulatory genes is illustrated in Fig. 4, in which the duplicated paralogs are listed together. The expression levels of duplicated paralogs were highly variable. Some paralogs showed extremely low expression levels or expression was not detected, which indicated that these duplicated genes showed functional redundancy and only a portion of paralogs play roles in anthocyanin biosynthesis. Examples are the five *F3H* genes (*BoF3H1–5*) and the two *BoDFRs* that have been mentioned previously.

Most of upstream genes (phenylpropanoid pathway genes and EBGs) did not show differential expressions in different leaves, which indicated that these genes did not play important roles for anthocaynin accumulation differences. The LBGs, such as *BoDFR1*, *BoANS1*, *BoANS2* and *BoUGT79B1.1*, showed differential expression levels in new and mature leaves of ‘05-DH-65’ and ‘06-DH-71’. These genes all showed the highest expression levels in new leaves of ‘05-DH-65’, second-highest levels in mature leaves of ‘05-DH-65’, and low expression levels in new and mature leaves of ‘06-DH-71’, which are white and green and contain almost no anthocyanins (Fig. 4).

The expression of structural genes in the anthocyanin biosynthetic pathway is mainly regulated by R2R3-MYB, basic helix-loop-helix (bHLH) and WD40-type transcriptional factors and their interaction [14]. Some R2R3-MYB proteins, such as MYB11, MYB12, and MYB111, which independently regulate EBGs [31], showed relatively low expression levels in the four leaf types and mostly did not show significant differences in expression level; only *BoMYB111.1* was more highly expressed in ‘06-DH-71’ leaves than in ‘05-DH-65’ leaves (Fig. 4). The LBGs are mainly activated by MBW ternary transcriptional complexes [8]. Of the four R2R3-MYB genes (*Bol012528*, *Bol012531*, *Bol045347*, and *Bol042409*) that form the MBW complex, *Bol012531* showed extremely low expression levels in all four leaf types; *Bol042409* showed a higher expression level in ‘06-DH-71’ leaves than in ‘05-DH-65’ leaves; *Bol045347* showed the lowest expression level in new leaves of ‘05-DH-65’; *Bol012528* was more highly expressed in ‘05-DH-65’ leaves than in ‘06-DH-71’ leaves, with the highest expression level observed in mature leaves of ‘05-DH-65’ (Fig. 4). Four of the five bHLH coding genes, namely *BoGL3*, *BoEGL3.1*, *BoEGL3.2*, and *BoEGL3.3*, showed extremely low expression levels in all four leaf types, whereas *BoTT8* was more highly expressed in ‘05-DH-65’ leaves than in ‘06-DH-71’ leaves. The WD40 protein coding gene, *BoTTG1*, also showed a higher expression level in ‘05-DH-65’ leaves than in ‘06-DH-71’ leaves (Fig. 4).

The expression patterns of negative regulatory genes in new and mature leaves of ‘05-DH-65’ and ‘06-DH-71’ are shown in Additional file 1: Figure S3. Several transcription factors, including two R3-type single MYB proteins, *MYBL2* and *CPC*, and three N/NO_3_^−^ induced members of the *LBD* gene family, act as negative regulators of anthocyanin biosynthesis in *A. thaliana*. MYBL2 is a transcriptional repressor that negatively regulates anthocyanin biosynthesis by interacting with TT8 and MBW complexes (Dubos et al., 2008; [22]). *BoMYBL2.1* was only expressed in new and mature leaves of ‘06-DH-71’. *BoMYBL2.2* was expressed in all four leaf types and the expression level was slightly higher in new leaves. CPC, a single-repeat R3-MYB transcription factor, functions as a negative regulator of anthocyanin biosynthesis [43]. *BoCPC1*, *BoCPC2* and *BoCPC3* were all expressed very low in all samples. LBD37, 38, and 39 negatively regulate the late anthocyanin-specific steps by repressing PAP1 and PAP2 under N/NO_3_^−^ induction [29]. The *BoLBD* genes all showed the highest expression levels in new leaves of ‘05-DH-65’ and were highly expressed in new leaves than mature leaves.

*BoTT19.1* and *BoTT19.2*, the two TT19 coding genes, were most highly expressed in new leaves of ‘05-DH-65’, second highest in mature leaves of ‘05-DH-65’, followed by new leaves of ‘06-DH-71’, and showed the lowest expression level in mature leaves of ‘06-DH-71’. The FPKM value of *BoTT19.1* was ~ 380-fold higher in new leaves of ‘05-DH-65’ than in new leaves of ‘06-DH-71’ (Additional file 4: Figure S4).

Based on these results, the expressed levels of genes in anthocyanin biosynthetic pathway from different leaf color samples of ornamental kales were intuitively demonstrated (Fig. 4).

### Significant differentially expressed anthocyanin biosynthetic genes between red- and white-leaved accessions

**Fig. 5:**
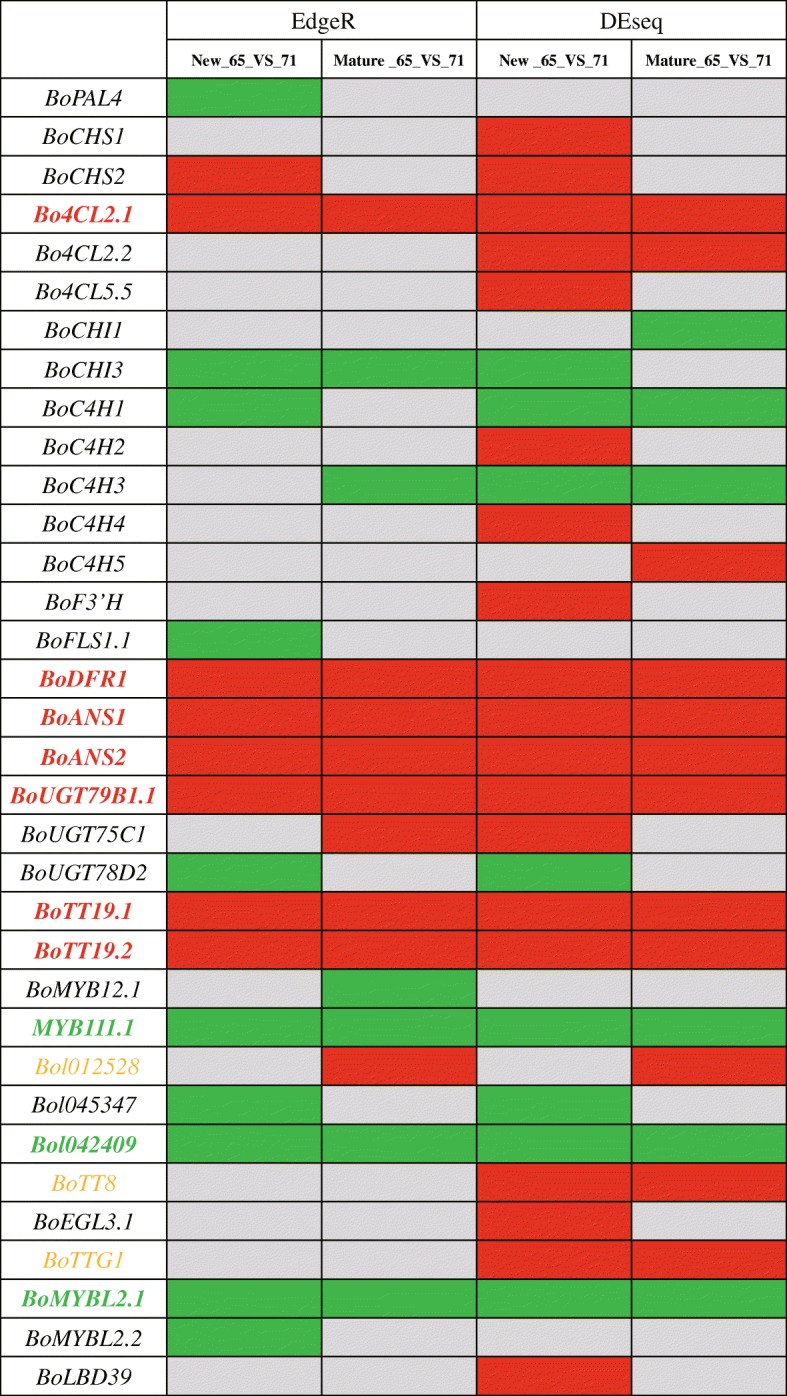
The significant differential expression ABGs in new and mature leaves between red accession ‘05-DH-65’ and white accession ‘06-DH-71’ by EdgeR and DEseq analysis. The red and green colors indicated significant up- and down-regulation of genes in new and mature leaves of ‘05-DH-65’, and the gray ones showed on significant expression difference

In order to analyze the significant up- and down-regulated BoABGs in red and white leaves of different accessions, the differentially expressed BoABGs were detected in new and mature leaves between red accession ‘05-DH-65’ and white accession ‘06-DH-71’ by software edgeR and DESeq2. The significant differentially expressed genes were selected by the threshold that fold change bigger than 2, *P* value and FDR (false discovery rate) less than 0.05 for edgeR, as well as fold change bigger than 2 and q value less than 0.05 for DESeq (Additional file 7: Table S4–9). The results of significant differentially expression analysis of anthocyanin biosynthetic genes in new and mature leaves between ‘05-DH-65’ and ‘06-DH-71’ were listed in Additional file 7: Table S4–9. And the significant up- and down- regulation genes were displayed in two volcano plots by EdgeR analysis (Additional file 5: Figure S5 and Additional file 6: Figure S6). From the results of significant differential genes expression analysis, we found that *Bo4CL2.1*, *BoANS1* and *2*, *BoDFR1*, *BoUGT79B1.1*, *BoTT19.1* and *19.2* were significantly up-regulated both in new and mature leaves of red accession ‘05-DH-65’ by EdgeR and DESeq2 analysis (Fig. 5, Additional file 7: Table S4–9). *BoDFR1*, *BoANS1* and *2*, and *BoUGT79B1.1* were LBGs leading to biosynthesis anthocyanins. *BoTT19.1* and *19.2* were anthocyanin transport genes functioning as carriers which played key roles in anthocyanin transportation and accumulation. The significant up-regulation of these two genes in new and mature leaves of red accession (Fig. 5) might be the essential factor for anthocyanin accumulation in leaves of ornamental kale. For the three MYB genes (*Bol012528*, *Bol012347* and *Bol042409*) which formed MBW complex activating LBGs, only *Bolo12528* was up-regulated in mature leaves of red accession by EdgeR and DESeq2 analysis, while the other two genes were down regulated. *BoTT8* and *BoTTG1* were both up-regulated in new and mature leaves of ‘05-DH-65’ by DESeq2 (Fig. 5). The MYB-bHLH-WD40 protein complex coding genes, *Bol012528*, *BoTT8* and *BoTTG1*, were up-regulated in red accession might play key positive regulatory roles to active LBGs, such as *BoDFR1*, *BoANS1* and *2*. The negative regulation gene *BoMYBL2.1* was significantly down-regulated in new and mature leaves of ‘05-DH-65’ by EdgeR and DESeq2 analysis, indicating its negative regulatory role in anthocyanin biosynthesis of ornamental kale. *MYB111.1* and *Bol042409* were also down-regulated in new and mature leaves of red accession (Fig. 5), and they might not play as positive regulators of anthocyanin biosynthesis in ornamental kale anymore.

In summary, the significant up-regulation LBGs: *BoDFR1*, *BoANS1* and *2*, regulatory genes: *Bol012528*, *BoTT8* and *BoTTG1*, and transport genes: *BoTT19.1* and *19.2*, as well as significant down-regulation negative regulatory gene: *BoMYBL2.1*, played roles in anthocyanin biosynthesis and accumulation in red ornamental kale.

## Discussion

### Variation in leaf color of ornamental kale

Ornamental kales, which change leaf color from green to red or white under low temperature because of differences in anthocyanin accumulation capabilities, are ideal materials to study the genetic mechanism of anthocyanin biosynthesis of *B. oleracea*. Anthocyanins were accumulated in the epidermal cells and adjacent mesophyll cells in new leaves of a red-leaved accession, while almost no chlorophylls are present, resulting in the entirely red leaf phenotype. The mature leaves of the red-leaved accession contained a reduced anthocyanins content, but higher amounts of chlorophylls resulting in the green lamina, with only leaf veins and the petiole containing low amounts of chlorophylls exhibiting a light red color. However, the new leaves of the white-leaved accession contained almost no pigments (Fig. 1). The observations showed that the pigment distribution in the different leaves was consistent with the color phenotype, and demonstrated that red pigments were mainly located in the epidermal cells of ‘05-DH-65’ and new leaves contained a higher amount of red pigment than mature leaves. However, almost no pigments were visible in ‘06-DH-71’ new leaves. Thirty-four anthocyanins were identified in the red-leaved ornamental kale. Cyanidin glycosides are the most common anthocyanins reported in *Brassica* crop species [10, 18, 30]. It is interesting that one pelargonidin and four delphinidin glycosides were identified, whereas only cyanidin glycosides were detected in Zicaitai (*Brassica rapa* L. ssp. *chinensis* var. *purpurea*) [10], but further verification against standards is needed. The TAC was ~ 20-fold higher in new leaves than in mature leaves of the red-leaved accession, whereas almost no anthocyanins were detected in the white-leaved accession (Fig. 2). The present phenotypic assessment and anthocyanins profile and contents analysis illustrated the relationship between the color phenotype and anthocyanin accumulation of the leaves, and provides a foundation to elucidate the genetic mechanism of leaf coloration and anthocyanin biosynthesis in *B. oleracea*.

### Anthocyanin biosynthetic genes expanded by WGD and TD in *B. oleracea*

Eighty-one ABGs were identified in the *B. oleracea* genome, which was almost double the number in the *A. thaliana* genome. Whole genome duplication and TD represent the major mechanisms for BoABGs expansion. Some upstream structural genes (phenylpropanoid pathway genes and EBGs) have been expanded through TD to form gene clusters in *B. oleracea*, such as C4H, 4CL, F3H, and FLS coding genes (Table 2). The tandem arrays of *C4H*, *4CL*, and *FLS* are also present in the *B. rapa* genome [9]. These results demonstrated that upstream structural genes expanded by TD in *Brassica* species was important for biosynthesis of flavonoid compounds, such as anthocyanins and flavonols. Based on the presence of these homologous structural genes, the anthocyanin biosynthetic pathway in *B. oleracea* was then established. The regulatory genes were mainly expanded by WGD. Some negative regulators, such as *CPC*, *LBD37*, and *LBD38*, retained three copies in different subgenomes (Table 2). From the present expression data, we speculate that these negative regulators might not play important negative regulatory roles in anthocyanin biosynthesis of ornamental kale (Additional file 3: Figure S3). These genes might have undergone neo- or sub-functionalization after WGD. The distribution analysis showed that some BoABGs were mapped to different locations of the two *B. oleracea* reference genome versions (Fig. 3). A higher number of BoABGs were mapped on the genome V2.1 (TO1000), which indicated that the assembly of TO1000 was more complete. The differences in distribution of BoABGs might also reflect assembly errors or structural variations of the two reference genome sequences, so additional complete and accurate reference genomic and pan-genomic sequences are essential for *B. oleracea*. The distribution analysis may provide a basis for further genetic mapping and molecular breeding studies. Identification of the complete set of anthocyanin biosynthetic genes in the *B. oleracea* genome provides a valuable resource for investigations of genetic mechanisms.

### Differential expression of BoABGs determines anthocyanin accumulation in ornamental kales

The higher expression levels of upstream biosynthetic genes in new leaves of the ornamental kale accessions provides the capacity to synthesize higher quantities of flavonoid compounds, such as anthocyanins and flavonols. In leaves, flavonoids are usually considered to function as antioxidants [38]. We present the following hypotheses. Under low temperature, flavonoid biosynthesis might be induced to accumulate flavonoid compounds in ornamental kale to protect the new leaves from intracellular damage caused by enhanced accumulation of reactive oxygen species. Given the genetic variation, different genotypes would synthesize and accumulate different flavonoids; thus, red-leaved accessions may accumulate anthocyanins whereas colorless flavonoids would accumulate in white-leaved accessions that are unable to synthesize anthocyanins. The LBGs leading to anthocyanins biosynthesis, such as *BoDFR1*, *BoANS1* and *2*, and *BoUGT79B1.1*, were most highly expressed in the new leaves of the red-leaved accession, and second highest in mature leaves of the red-leaved accession, and showed an extremely low expression level in the leaves of the white-leaved accession. The expression patterns of these LBGs in the different leaf types were in accordance with the anthocyanins contents and leaf color phenotypes. These genes may play vital roles in anthocyanin biosynthesis in ornamental kale.

The positive regulatory genes *Bol012528*, *BoTT8*, and *BoTTG1*, which form the MBW transcription complex to activate LBGs, such as *BoDFR1* and *BoANS1* and *2*, positively regulate anthocyanins biosynthesis in ornamental kale. The negative regulatory gene *BoMYBL2.1* was only expressed in leaves of the white-leaved accession ‘06-DH-71’, which would repress LBGs expression in white and green leaves to prevent anthocyanins biosynthesis. Other negative regulatory genes, such as *BoLBDs*, which showed the higher expression levels in new leaves, did not play negative roles in anthocyanin biosynthesis of ornamental kale. *BoMYBL2.1* was indicated to play the main negative regulatory role in anthocyanin accumulation in ornamental kale.

TT19 is a carrier protein for sequestering anthocyanin into the vacuole. The *TT19* mutant of *Arabidopsis* with no visible anthocyanins accumulated in cotyledons and hypocotyls of seedlings grown on the anthocyanin induction media demonstrated the key role of *TT19* in anthocyanin accumulation and coloration [32]. *BoTT19.1* and *BoTT19.2* showed a significantly higher expression level in red leaves than in white and green leaves. The expression patterns of these genes were consistent with the patterns of anthocyanins accumulation and color phenotypes of different leaves of red- and white-leaved ornamental kale accessions. The DEGs analysis also demonstrated that *BoANS1* and *2*, *BoDFR1*, *BoUGT79B1.1*, *BoTT19.1* and *19.2* were significantly up-regulated in red accession, while *BoMYBL2.1* was significantly up-regulated in white accession (Fig. 5). Thus, these genes are indicated to play vital roles in anthocyanins biosynthesis and accumulation in ornamental kale.

### Genetic network of anthocyanins accumulation in ornamental kale

**Fig. 6:**
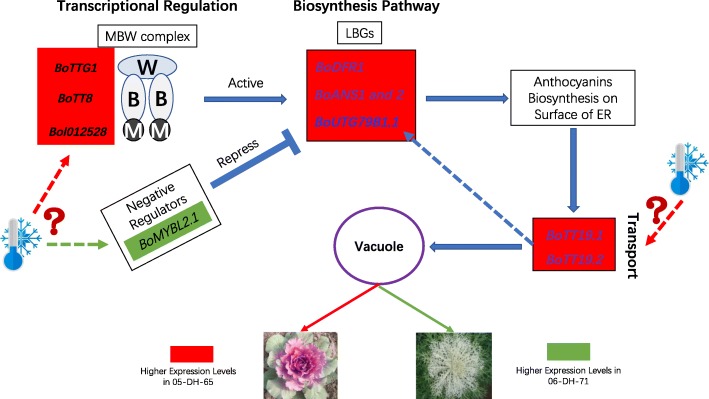
Regulatory network of anthocyanins accumulation in red- and white-leaved ornamental kales. In red leaves, *BoTTG1*, *BoTT8*, and *Bol012528* form the MBW complex to activate *BoDFR1*, *BoANS1* and *2*, *BoUGT79B1.1*, and *BoUT78D2* to promote anthocyanins biosynthesis on the cytosolic surface of the ER. In white and green leaves, the negative regulator *BoMYBL2.1* is highly expressed to repress the LBGs. The transporter genes *BoTT19.1* and *19.2* are highly expressed in red leaves to transport anthocyanins from the ER to the vacuole, thereby promoting the red leaf phenotype. High expression levels of *BoTT19.1* and *19.2* may also feedback to activate LBGs by transporting greater amounts of anthocyanins

Under low temperature, the new leaves of different ornamental kale genotypes will exhibit red and white colors because of inhibition of chlorophyll synthesis and variation in anthocyanins accumulation capacities. To elucidate the genetic mechanism of anthocyanin biosynthesis and leaf color variation of ornamental kales, a comparative whole-genome identification and expression analysis of anthocyanins between red- and white-leaved accessions of BoABGs was conducted. Based on these results, a metabolic pathway and regulatory network was constructed to represent anthocyanins biosynthesis and accumulation in ornamental kales (Fig. 6). The positive regulatory genes *Bol012528*, *BoTT8*, and *BoTTG1* forming the MBW complex were up-regulated in the red-leaved accession to activate the LBGs, especially *BoDFR1*, *BoANS1* and *2*, and *BoUGT79B1.1,* which lead to anthocyanins synthesis. In contrast, these positive regulatory genes were down-regulated and a negative regulatory gene, *BoMYBL2.1*, was up-regulated in the white and green leaves of the white-leaved accession, thereby repressing the LBGs to inhibit anthocyanin biosynthesis. The three MBW complex-forming positive regulatory genes and one negative regulatory gene functioned as transcriptional factors to regulate anthocyanin biosynthesis in ornamental kale. The anthocyanins transport genes *BoTT19.1* and *19.2*, which were almost only expressed in red leaves, would play vital roles in anthocyanins accumulation in ornamental kale. This genetic regulatory network of anthocyanins accumulation in different accessions explains the variation in leaf color and enhances the understanding of leaf color development under low temperature in ornamental kale. These findings pave the way for further genetic modification of leaf color and the breeding of novel cultivars of *B. oleracea*.

## Conclusions

We determined pigments distributions in red- and white-leaved accessions which could explain the leaf color phenotypes of ornamental kales. Thirty-four anthocyanins were detected in the red-leaved accession. Eighty-one anthocyanin biosynthetic genes were identified in the *B. oleracea* reference genome. The expression patterns of these genes in different leaf types indicated that late biosynthetic genes (*BoDFR1*, *BoANS1* and *2* and *BoUGT79B1.1*), positive regulatory genes (*BoTTG1*, *BoTT8*, and *Bol012528*), a negative regulatory gene (*BoMYBL2.1*), and transport genes (*BoTT19.1* and *BoTT19.2*) play key roles in anthocyanin accumulation in ornamental kale. A genetic regulatory network of anthocyanin accumulation in ornamental kale was constructed. These results enhance the understanding of the genetic mechanisms and regulatory network of anthocyanin accumulation in *B. oleracea* under low temperature, and provide a theoretical basis for breeding new cultivars of *Brassica* vegetables with increased anthocyanin contents and cold resistance.

## Methods

### Plant materials, phenotypic characterization, and leaf anatomical observations

Two ornamental kale doubled-haploid (DH) lines (produced by vegetable biotechnology group, Beijing Vegetable Resarch Center), ‘05-DH-65’ and ‘06-DH-71’, which differ in leaf color and shape, were selected for phenotype assessment, anthocyanins profiling, and RNA-seq analysis. ‘05-DH-65’ has entire leaves and the central leaves are red under low temperature (Fig. 1a), whereas ‘06-DH-71’ has feather-like divided leaves and the central leaves change in color from green to white under low temperature (Fig. 1b). The plant materials were grown in an unheated research greenhouse of the National Engineering Research Center for Vegetables, Beijing, China, during the 2016–2017 cropping season. The two DH lines were cultivated side by side each with three replications. Central (young) and outer (mature) leaves were collected at the rosette stage after color development (at temperatures under 10 °C) for observation of leaf anatomy, analysis of anthocyanin contents and profiles, and RNA extraction for RNA-seq analysis. The phenotype of the central and outer leaves from ‘05-DH-65’ and ‘06-DH-71’ was compared. In addition, to examine purple pigment accumulation, portions of the lamina and petiole were transversely and longitudinally sectioned, respectively, by hand and examined with a Nikon TS100 microscope.

### Anthocyanins content determination by pH differential spectrophotometry

The pH differential spectrophotometry method was performed in accordance to that reported by [15], with slight modifications [10], to determine total anthocyanins content (TAC) in central and outer leaves of ‘05-DH-65’ and ‘06-DH-71’. Absorbance was measured at 520 and 700 nm. The TAC was expressed as cyanidin-3-glucoside (cyd-glu, molar extinction coefficient 26,900 l/cm mol, molecular weight 449.2 g/mol) equivalents. The units for TAC were mg/g dry weight (DW) of the detected sample. For all samples, TAC measurements were replicated three times.

### Anthocyanin profile characterization

The chromatographic system, which was set up in accordance with the method described previously [10] with modifications, which consisted of a Shimadzu LC-30A UHPLC system equipped with an ultraviolet detector. Chromatographic separation was achieved using a Waters ACQUITY UPLC™ BEH C18 (2.1 × 100 mm, 1.7 μm) at a flow rate of 0.3 ml/min. The column oven temperature was set at 40 °C. The mobile phase was a binary solvent system consisting of solvent A (formic acid 0.1% [v/v] in water) and solvent B (acetonitrile). The gradient conditions were as follows: 5–15% B from 0 to 25 min, maintained at 15% B for 20 min; 15–18% B from 45 to 60 min; 18–95% B from 60 to 70 min, maintained at 95% B for 2 min; and 5% B from 72 to 75 min for equilibration of the column for the next run. The sample injection volume was 3 μL. Chromatograms were recorded at 520 nm for anthocyanins. The analytes were identified using an AB SCIEX TripleTOF® 6600 system equipped with a Turbo V™ ion source. Product ion spectra were acquired using time-of-flight mass spectrometry and information-dependent acquisition compound scan modes. The data acquisition used Analyst® TF 1.7.1 software, and PeakView® 2.1 and MasterView™ software were used for data analysis.

### Identification of anthocyanin biosynthetic genes by comparative genomic analysis

Anthocyanin biosynthetic genes of *B. oleracea* were identified by syntenic and non-syntenic homolog analysis with *A. thaliana*. The reference genome of *B. oleracea* we used for anthocyanin biosynthetic genes identification was Cap02–12 [19]. We identified syntenic orthologs between *A. thaliana* and *B. oleracea* from the *Brassica* Database (BRAD; http://brassicadb.org/brad/) using both sequence similarities (cutoff: E ≤ 1E− 20) and the collinearity levels of flanking genes [7]. The homologous relationships were analyzed by BLASTN and BLASTP algorithm-based searches using gene and protein sequences, respectively, with a cut off E-value ≤1E− 10 and coverage ≥0.75 [9]. Then we identified the homolog genes in another reference genome TO1000 [24], and analyzed the chromosomes locations of these genes on the two references.

### RNA extraction

Total RNA was extracted using TRIzol™ reagent (Invitrogen, 15,596–026). The contaminant DNA was removed using the TURBO DNA-free™ Kit (Ambion, AM1907). The quality of purified RNA was initially evaluated on agarose gel and then quantified using a NanoDrop™ spectrophotometer (Thermo Fisher Scientific, Inc.). The integrity of RNA samples was further evaluated using an Agilent 2100 Bioanalyzer (Agilent Technologies, Inc.).

### Library construction and sequencing

The TruSeq™ RNA Sample Preparation Kit (Illumina, Inc.) was used to construct cDNA libraries in accordance with the manufacturer’s instructions. Briefly, poly-A mRNA was purified and fragmented into short fragments and used as templates for first-strand cDNA synthesis. DNA polymerase I and RNase H were used to synthesize the second-strand cDNA. Purified short double-strand cDNA fragments were connected with adapters (Illumina). Suitable ligated cDNA fragments were selected as templates for PCR amplification for final library construction. Finally, the cDNA libraries were sequenced using the Illumina HiSeq® 2500 platform.

### RNA-seq data analysis

Adaptors were removed from the RNA-Seq reads. The reads in which unknown bases comprised more than 5% of the total and low-quality reads (the percentage of the low-quality bases of quality value ≤5 is more than 50% in a read) were also removed. The clean reads were aligned to the cabbage genome (*B. oleracea* var. *capitata* line 02–12) [19] accessed from BRAD [5], allowing up to two mismatches in each segment alignment by TopHat [34, 35] and Bowtie software. Only unique mapped reads were used for further analysis.

Gene expression level and transcript abundances were calculated using the FPKM (fragments per kilobase of transcript per million mapped reads) method [36]. The FPKM values of anthocyanin biosynthetic genes from leaves of the different ornamental kales were log2 transformed (in order to avoid the minus value after transformation, we added “1” to every FPKM values before log2 transformation), mean centered, and further used for hierarchical clustering and heatmap generation with the pheatmap package in R software.

### Differential gene expression analysis

The differential expression analysis was carried out by using the Bioconductor package EdgeR (Empirical Analysis of Digital Gene Expression Data in R) (Robinson et al., 2010 and McCarthy et al., 2012) and DESeq2 package [1]. The significant differential expression genes were selected by the threshold that fold change bigger than 2, P_value and FDR (false discovery rate) less than 0.05.

## Additional files

Additional file 1: **Figure S1.** (A) The expression values distribution of different samples demonstrated the overall expression levels among different samples. (B) A cluster dendrogram showed biological replicates sampled with good agreement replicates which was analyzed by gene expression values between each other. (PDF 813 kb)

Additional file 2: **Figure S2.** Heatmap representing expression profiles of anthocyanin biosynthetic genes in new and mature leaves of the ornamental kale DH lines ‘05-DH-65’ and ‘06-DH-71’. Blue and red colors are used to represent low to high expression levels, respectively. The color scale corresponds to the mean-centered log2-transformed FPKM values. (PDF 190 kb)

Additional file 3: **Figure S3.** Expression levels of negative regulatory genes involved in anthocyanin biosynthesis in new and mature leaves of the ornamental kale DH lines ‘05-DH-65’ and ‘06-DH-71’. (PDF 13 kb)

Additional file 4: **Figure S4.** Expression levels of the transport genes *TT19.1* and *TT19.2* in new and mature leaves of the ornamental kale DH lines ‘05-DH-71’ and ‘06-DH-71’. (PDF 252 kb)

Additional file 5: **Figure S5.** Volcano plot demonstrated significantly expressed BoABGs between new leaves of ‘05-DH-65’ and ‘06-DH-71’ by edgeR DEGs analysis. Green dots indicated the eight significantly up-regulated while red dots indicated the 11 significantly down-regulated BoABGs in new leaves of ‘05-DH-65’. (PDF 271 kb)

Additional file 6: **Figure S6.** Volcano plot demonstrated significantly expressed BoABGs between mature leaves of ‘05-DH-65’ and ‘06-DH-71’ by edgeR DEGs analysis. Green dots indicated the nine significantly up-regulated while red dots indicated the six significantly down-regulated BoABGs in mature leaves of ‘05-DH-65’. (PDF 230 kb)

Additional file 7: **Table S1.** The corresponding gene IDs of ABGs in the two reference genome versions of *B. oleracea*. **Table S2.** The FPKM values of ABGs in red- and white- leaf color accessions. (XLSX 77 kb)

## Data Availability

The plant materials as well as the raw data of their phenotypes and anthocyanin profiles during the current study are available from the corresponding author on reasonable request. The reference genome of *B. oleracea* Cap02–12 and TO1000 are available from Brassca Database (BRAD): http://brassicadb.org/brad/ and EnsemblPlants genome database: http://plants.ensembl.org/Brassica_oleracea/Info/Index, respectively. The RNA-seq datasets during the current study are available from the corresponding author on reasonable request. The expression datasets of antocyanin biosynthetic genes in red- and white-leaved ornamental kales are included in this published article.

## Abbreviations

4CL: 4-coumarate: CoA ligase
ABGs: Anthocyanin biosynthetic genes
ANS: Anthocyanidin synthase
AtABGs: Anthocyanin biosynthetic genes in *A. thaliana*
bHLH: Basic helix-loop-helix
BoABGs: Anthocyanin biosynthetic genes in *B. oleracea*
BrABGs: Anthocyanin biosynthetic genes in *B. rapa*
C4H: Cinnamate-4-hydroxylase
CHI: Chalcone isomerase
CHS: Chalcone synthase
CPC: CAPRICE
DFR: Dihydroflavonol-4-reductase
EBGs: Early biosynthetic genes
EGL3: Enhancer of glabrous 3
ER: Endoplasmic reticulum
F3′H: Flavonoid 3′-hydroxylase
F3H: Flavanone 3-hydroxylase
FLS: Flavonol synthase
FPKM: Fragments per kilobase of transcript per million mapped reads
GL3: Glabrous 3
GST: Glutathione S-transferase
LBD: Lateral organ boundary domain
LBGs: Late biosynthetic genes
LC–MS: Liquid chromatography–mass spectrometry
MBW: MYB-bHLH-WD40
MYBL2: MYB-like 2
PAL: Phenylalanine ammonia-lyase
PAP1/2: Production of anthocyanin pigment 1/2
TAC: total anthocyanins content
TD: Tandem duplication
TT8/19: Transparent testa 8/19
TTG1: Transparent testa glabrous 1
UFGT: UDP-glucose:flavonoid 3-O-glucosyltransferase
WGD: Whole genome duplication
WGT: Whole genome triplication
