# Supplementary figures and images for: Identification and differential expression analysis of anthocyanin biosynthetic genes in leaf color variants of ornamental kale
Source: BMC Genomics. 2019 Jul 8;20:564. doi: 10.1186/s12864-019-5910-z (PMC6615239; doi:10.1186/s12864-019-5910-z)

A

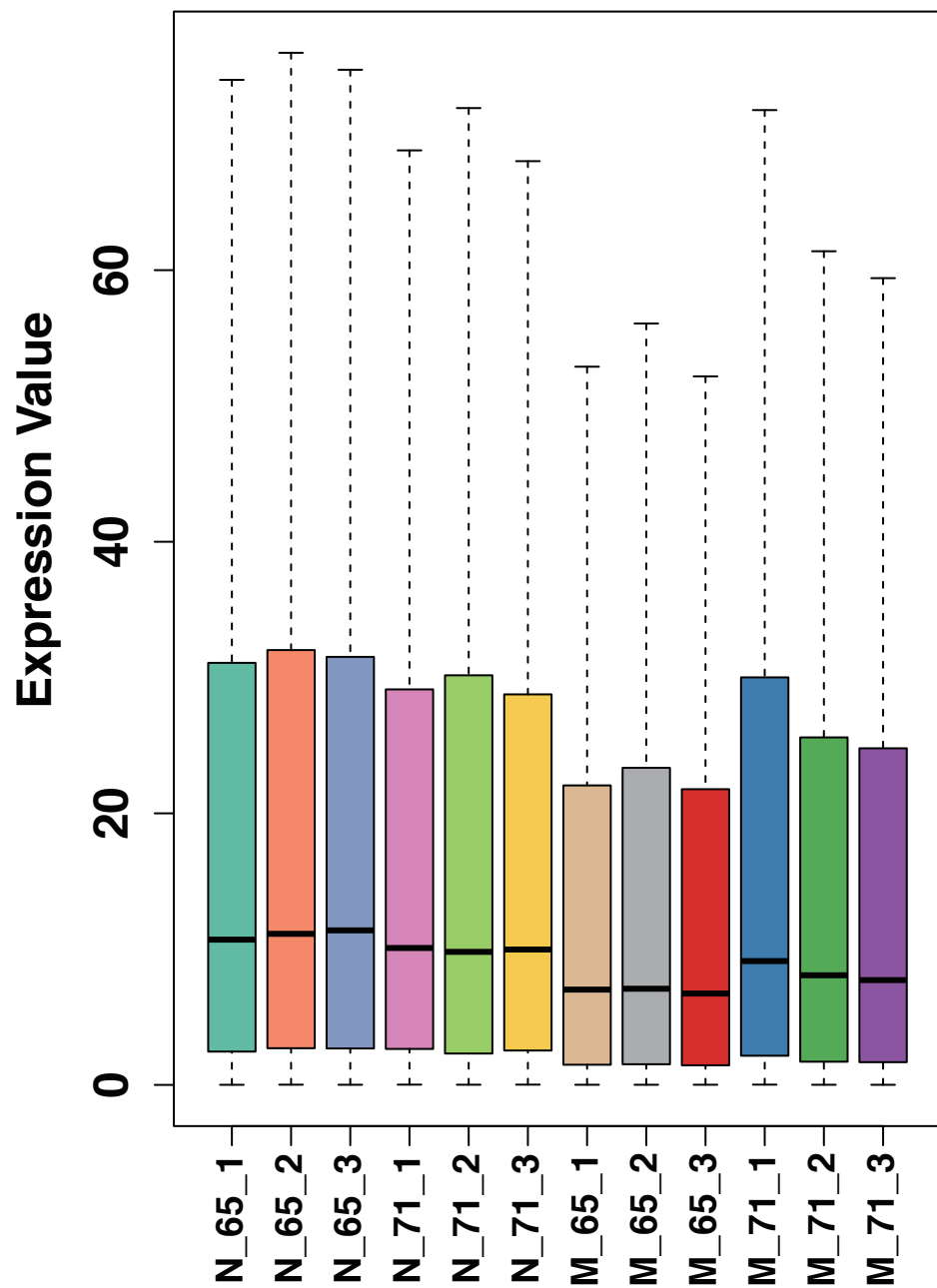

B

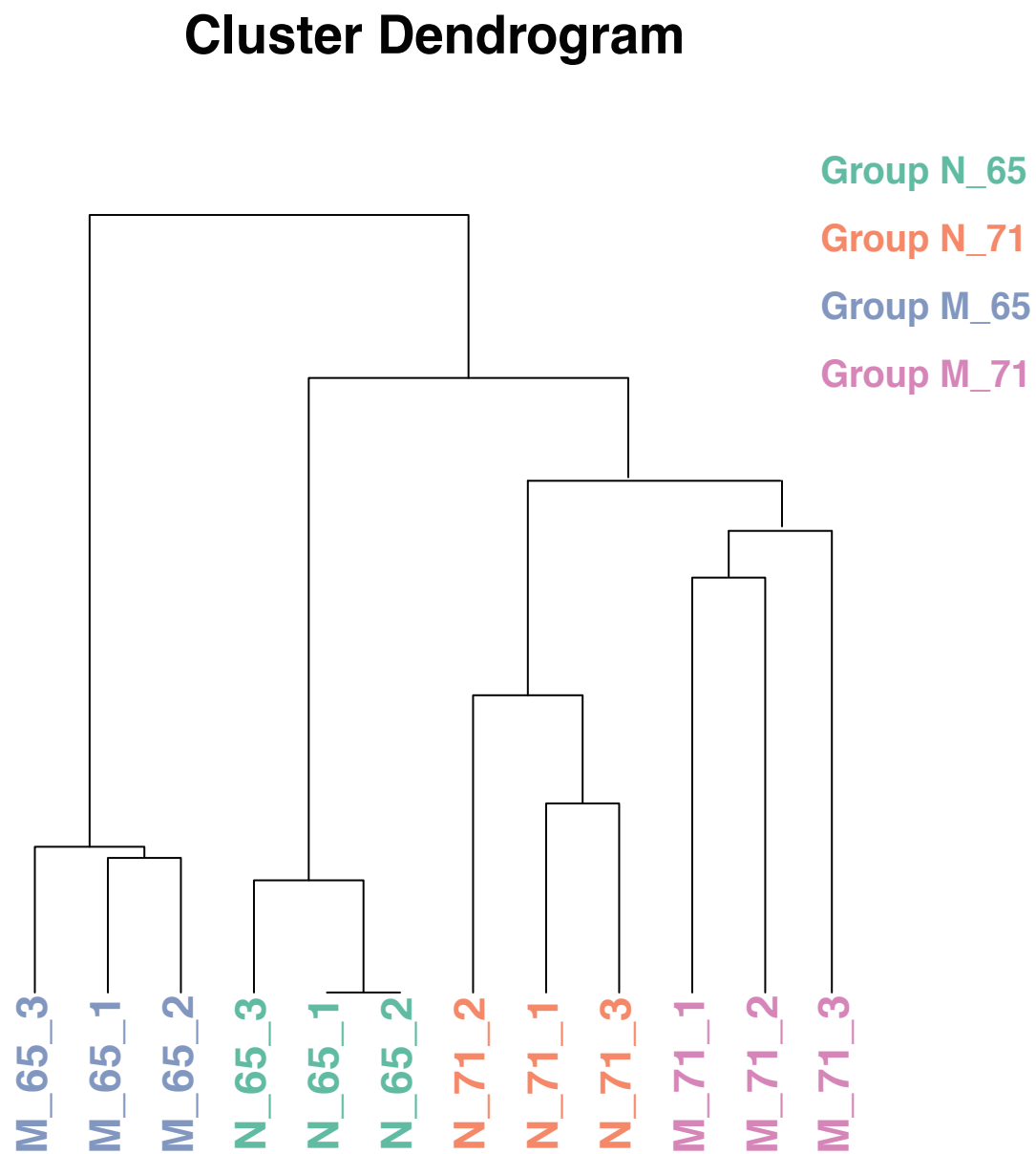

Supplement: Supplementary file 1 — Figure S1. (A) The expression values distribution of different samples demonstrated the overall expression levels among different samples. (B) A cluster dendrogram showed biological replicates sampled with good agreement replicates which was analyzed by gene expression values between each other. (PDF 813 kb) [file 12864_2019_5910_MOESM1_ESM.pdf]

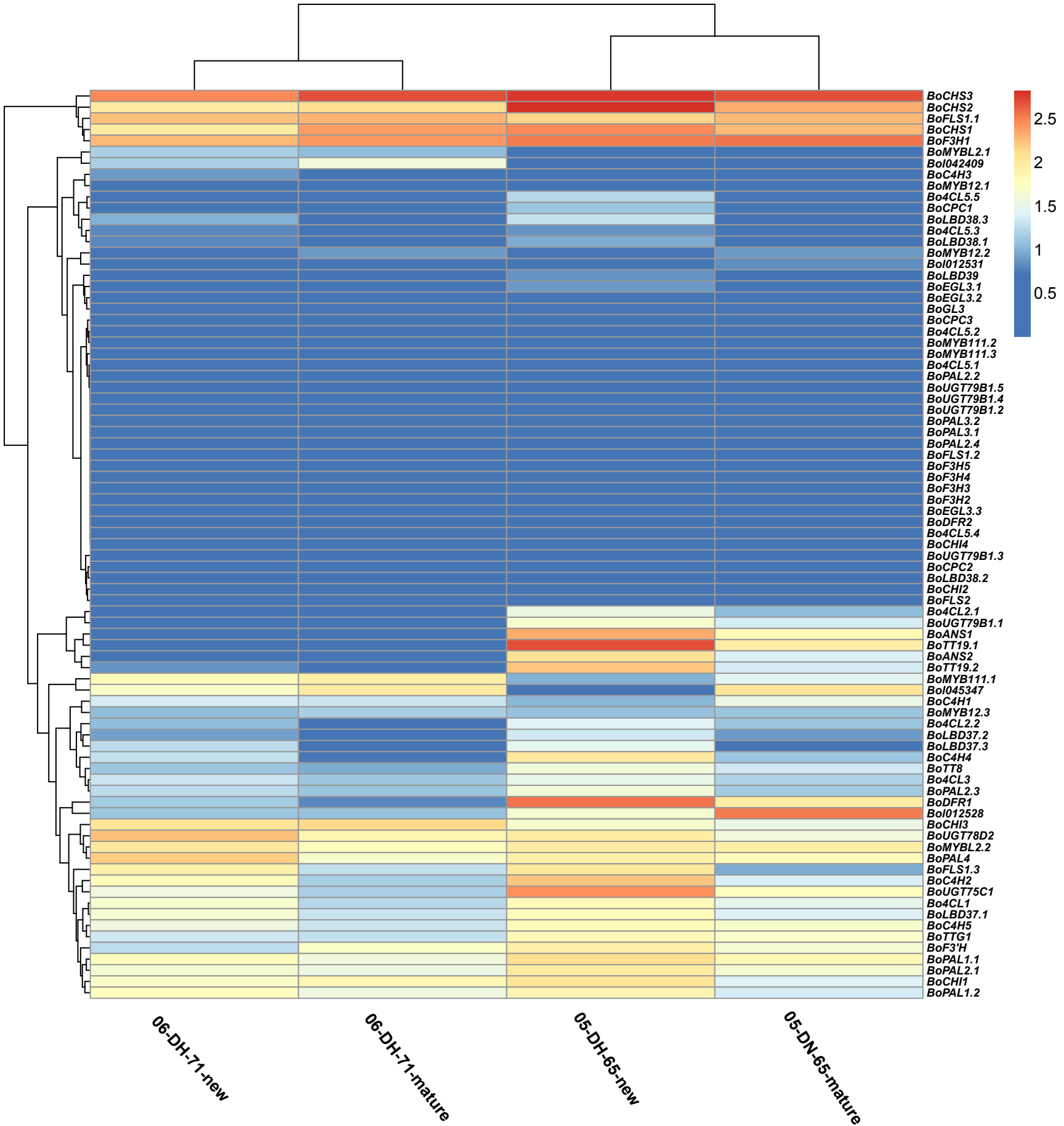

Supplement: Supplementary file 2 — Figure S2. Heatmap representing expression profiles of anthocyanin biosynthetic genes in new and mature leaves of the ornamental kale DH lines ‘05-DH-65’ and ‘06-DH-71’. Blue and red colors are used to represent low to high expression levels, respectively. The color scale corresponds to the mean-centered log2-transformed FPKM values. (PDF 190 kb) [file 12864_2019_5910_MOESM2_ESM.pdf]

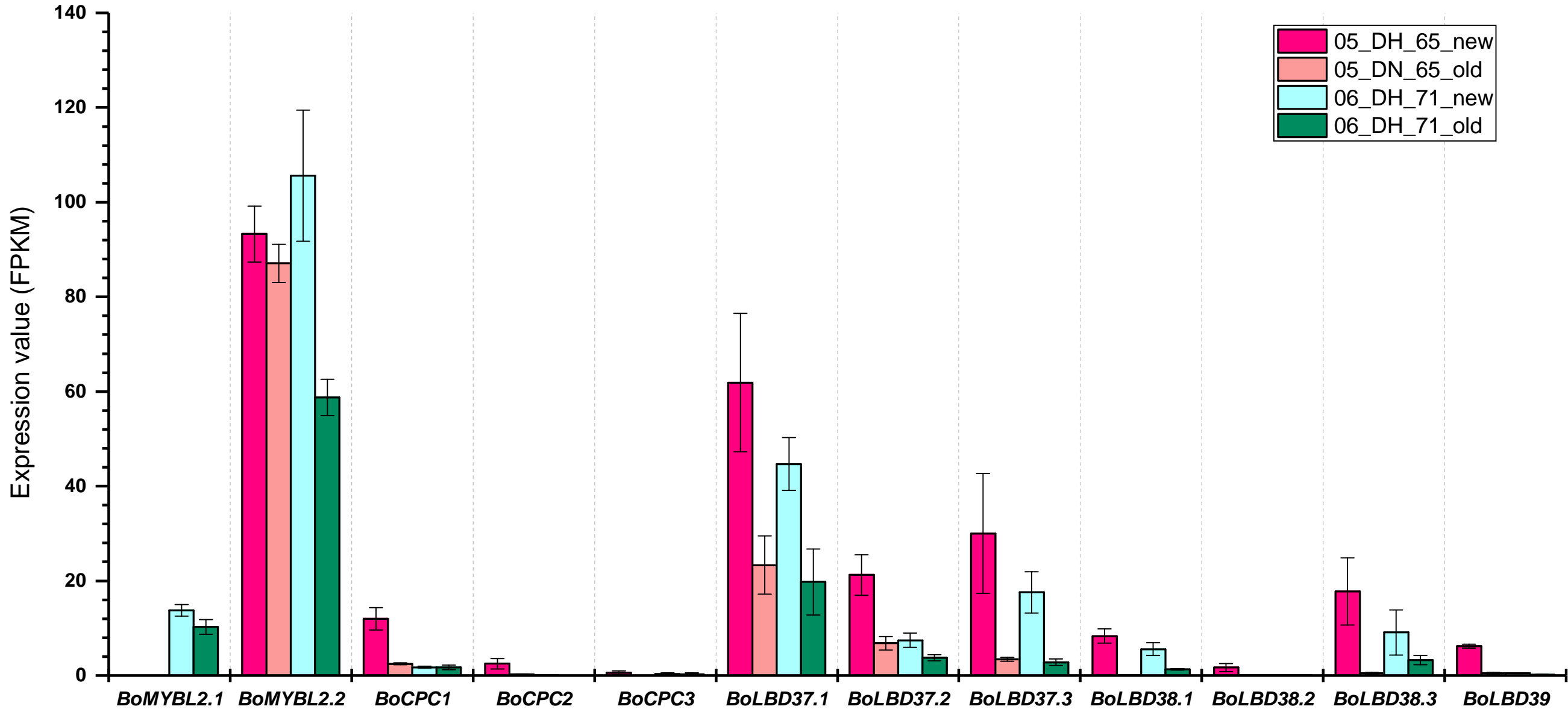

Supplement: Supplementary file 3 — Figure S3. Expression levels of negative regulatory genes involved in anthocyanin biosynthesis in new and mature leaves of the ornamental kale DH lines ‘05-DH-65’ and ‘06-DH-71’. (PDF 13 kb) [file 12864_2019_5910_MOESM3_ESM.pdf]

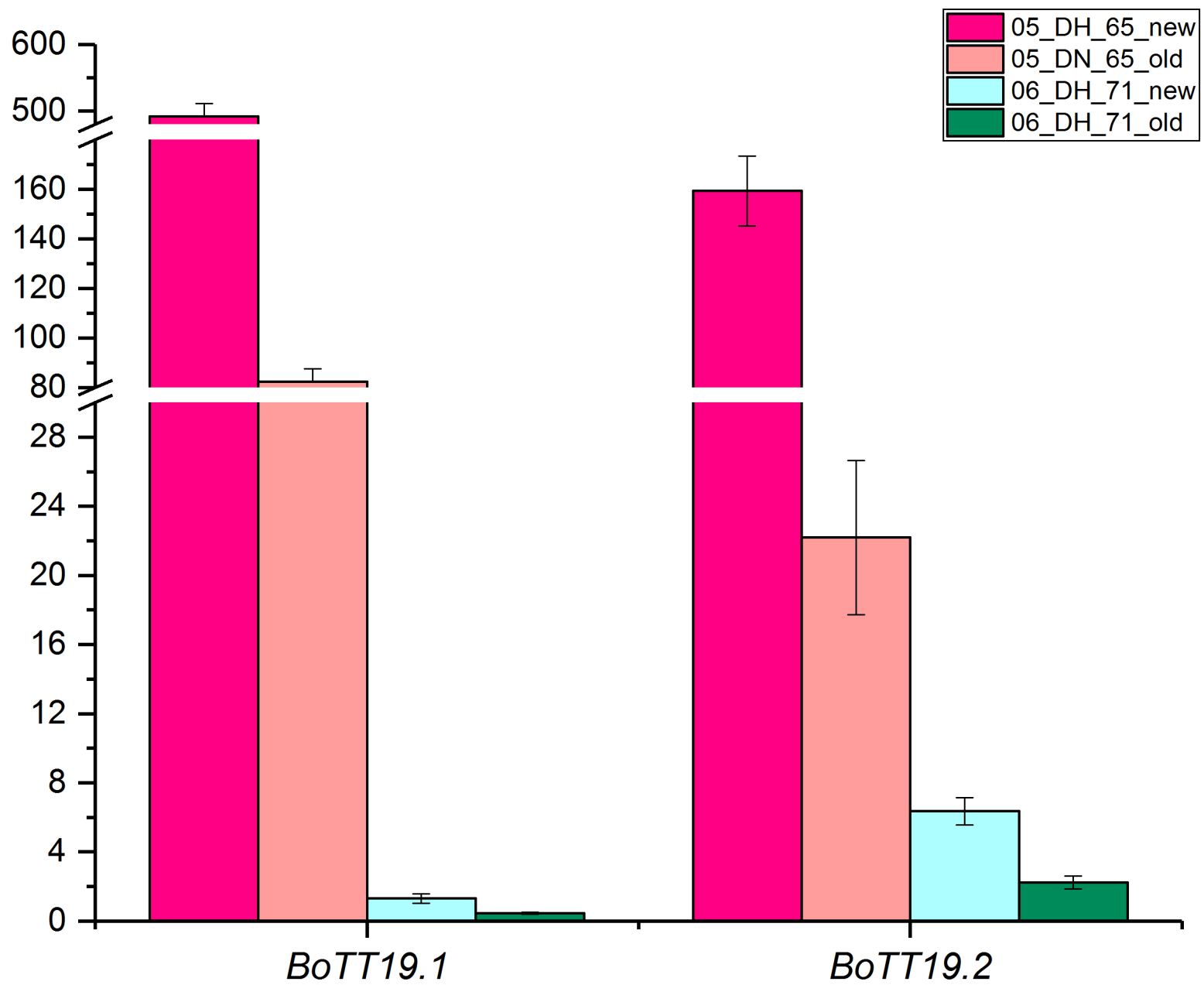

Supplement: Supplementary file 4 — Figure S4. Expression levels of the transport genes TT19.1 and TT19.2 in new and mature leaves of the ornamental kale DH lines ‘05-DH-71’ and ‘06-DH-71’. (PDF 252 kb) [file 12864_2019_5910_MOESM4_ESM.pdf]

05\_DH\_65\_new-vs-06\_DH\_71\_new volcano plot

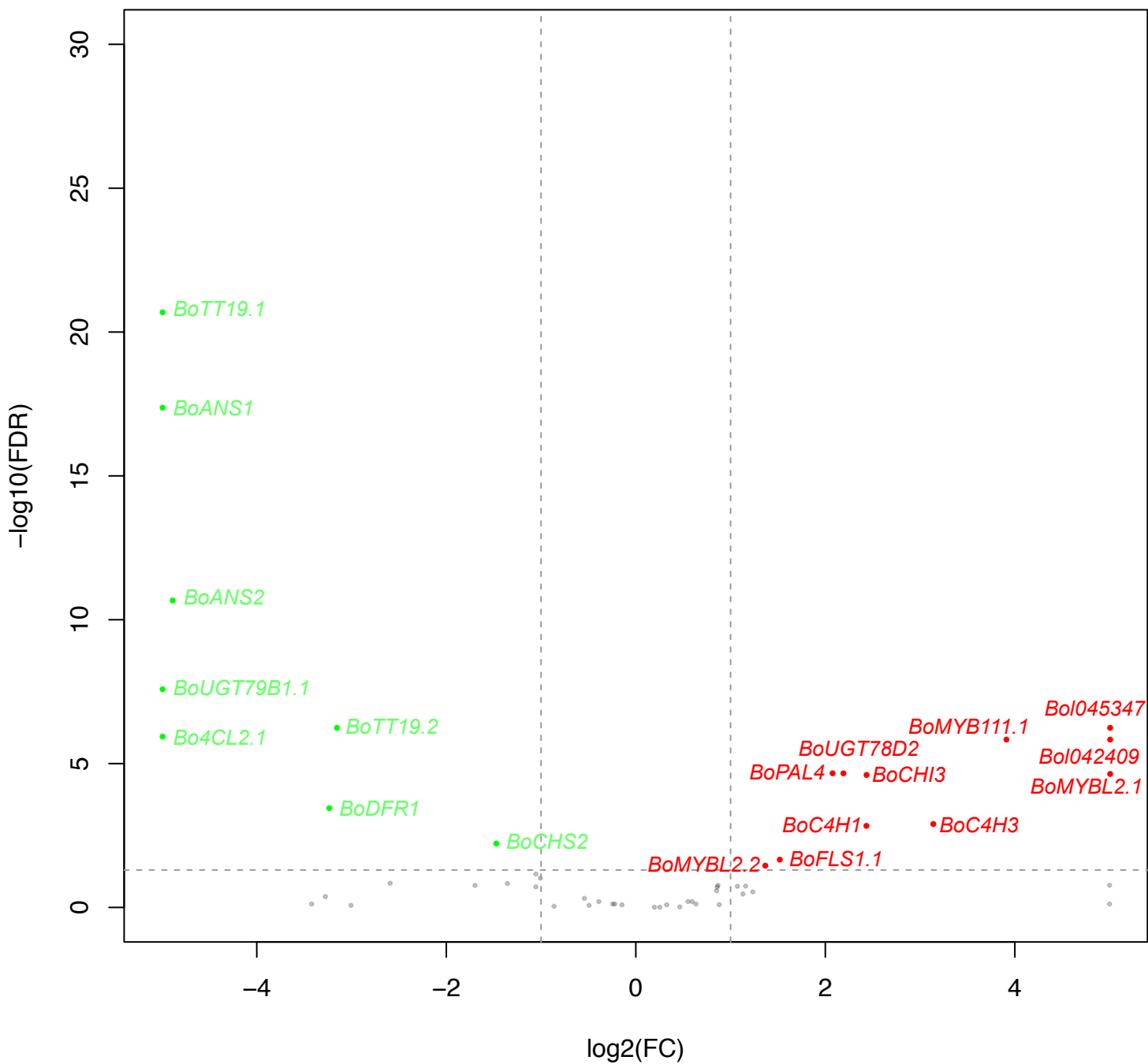

Supplement: Supplementary file 5 — Figure S5. Volcano plot demonstrated significantly expressed BoABGs between new leaves of ‘05-DH-65’ and ‘06-DH-71’ by edgeR DEGs analysis. Green dots indicated the eight significantly up-regulated while red dots indicated the 11 significantly down-regulated BoABGs in new leaves of ‘05-DH-65’. (PDF 271 kb) [file 12864_2019_5910_MOESM5_ESM.pdf]

05\_DN\_65\_mature-vs-06\_DH\_71\_mature volcano plot

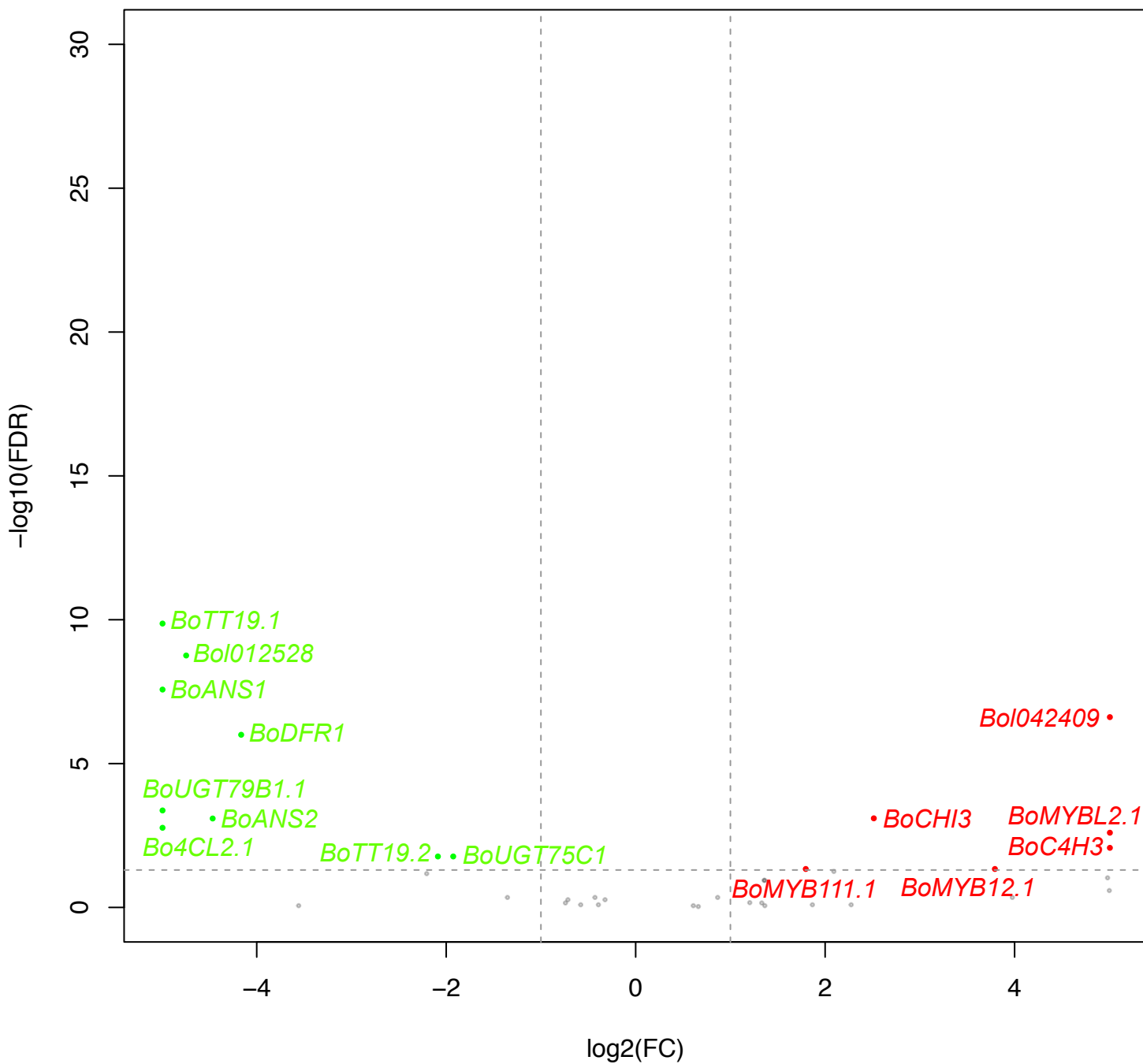

Supplement: Supplementary file 6 — Figure S6. Volcano plot demonstrated significantly expressed BoABGs between mature leaves of ‘05-DH-65’ and ‘06-DH-71’ by edgeR DEGs analysis. Green dots indicated the nine significantly up-regulated while red dots indicated the six significantly down-regulated BoABGs in mature leaves of ‘05-DH-65’. (PDF 230 kb) [file 12864_2019_5910_MOESM6_ESM.pdf]
